# RBPsuite: RNA-protein binding sites prediction suite based on deep learning

**DOI:** 10.1186/s12864-020-07291-6

**Published:** 2020-12-09

**Authors:** Xiaoyong Pan, Yi Fang, Xianfeng Li, Yang Yang, Hong-Bin Shen

**Affiliations:** 1 Institute of Image Processing and Pattern Recognition, Shanghai Jiao Tong University, and Key Laboratory of System Control and Information Processing, Ministry of Education of China, Shanghai, 200240 China; 2 Key laboratory of Carcinogenesis and Translational Research, Peking University Cancer Hospital, Beijing, 100142 China; 3 Department of Computer Science and Engineering, Shanghai Jiao Tong University, Center for Brain-Like Computing and Machine Intelligence, Shanghai, 200240 China

**Keywords:** Deep learning, RNA-binding proteins, Linear RNAs, Circular RNAs

## Abstract

**Background:**

RNA-binding proteins (RBPs) play crucial roles in various biological processes. Deep learning-based methods have been demonstrated powerful on predicting RBP sites on RNAs. However, the training of deep learning models is very time-intensive and computationally intensive.

**Results:**

Here we present a deep learning-based RBPsuite, an easy-to-use webserver for predicting RBP binding sites on linear and circular RNAs. For linear RNAs, RBPsuite predicts the RBP binding scores with them using our updated iDeepS. For circular RNAs (circRNAs), RBPsuite predicts the RBP binding scores with them using our developed CRIP. RBPsuite first breaks the input RNA sequence into segments of 101 nucleotides and scores the interaction between the segments and the RBPs. RBPsuite further detects the verified motifs on the binding segments gives the binding scores distribution along the full-length sequence.

**Conclusions:**

RBPsuite is an easy-to-use online webserver for predicting RBP binding sites and freely available at http://www.csbio.sjtu.edu.cn/bioinf/RBPsuite/.

## Background

RNA-binding proteins (RBPs) are involved in many biological processes, their binding sites on RNAs can give insights into mechanisms behind diseases involving RBPs [1]. Thus, how to identify the RBP binding sites on RNAs is very crucial for follow-up analysis, like the impact of mutations on binding sites. With high-throughput sequencing developing, there is an explosion in the amount of experimentally verified RBP binding sites, e.g. eCLIP [2] in ENCODE [3]. However, these CLIP-seq data still cannot provide the full view of the RBP binding landscape, it is because CLIP-seq relies on gene expression which can be highly variable between experiments. But these big data can serve as training data for machine learning models to predict missing RBP binding sites that may not be detected in some experiments. For example, GraphProt encodes a RNA sequence and structure in a graph [4], which is fed into a support vector machine to classify RBP bound sites from unbound sites. GraphProt can detect the binding sequence and structure preference of RBPs and further predict the RBP binding sites on any input RNAs. Considering that RBPs have difference binding preferences, the machine leaning-based methods train RBP-specific models; each model is trained per RBP.

Recently, deep learning-based methods have achieved remarkable results on predicting RBP sites [5, 6]. For example, DeepBind is the first method to train a convolutional neural network (CNN) [7] to predicting RBP binding preference [6]. Inspired by DeepBind, iDeep integrates multiple sources of features to predict RBP binding sites using a multi-modal deep learning, which consists of a CNN and multiple deep belief networks [8]. RBPs bind to RNAs by recognizing both the sequence and structure context. Thus, iDeepS trains a hybrid network with two CNNs and a long-short temporary memory (LSTM) network [9] to infer binding sequences and structure preferences of RBPs [10]. In iDeepS, two CNNs handle the sequence input and structure inputs, respectively and the LSTM learns the dependency between sequences and structures to improve prediction performance. Different from iDeepS, pysster encodes the sequence and structure in a one-hot encoded matrix based on an extended alphabet, which combines the sequence and structure alphabet [11]. DeepCLIP applies a similar network architecture consisting of a hybrid CNN and LSTM to predict RBP binding sites on RNAs [12] and the network architecture is similar to iDeepS. iDeepE trains a local CNN and a global CNN to predict RBP binding sites from sequences alone [13]. The binding mechanism of RBP binding circular RNAs (circRNAs) is different from that of linear RNAs, and thus the trained models on RBP binding linear RNAs cannot generalize well to circRNAs, CRIP is specially developed for predicting RBP binding sites on circRNAs by using a codon-based encoding schema and hybrid deep models [14].

There exist several online webservers for RNA-protein interaction prediction based on traditional machine learning models, e.g. omiXcore [15] and SMARTIV [16, 17]. omiXcore is an RBP-general method, which trains a non-linear algorithm on pooled RNA-protein interactions and accepts the proteins and large RNAs with a size between 500 and 20,000 as inputs. Considering that different RBPs have different binding specificities, the RBP-specific method in general is superior to the RBP-general method, as demonstrated in [13]. SMARTIV accepts a set of RNA sequences in BED format file as the input, and applies Hidden Markov Model (HMM) to find the enriched combined sequence and structure motifs from in vivo binding data. In addition, SMARTIV cannot predict RBP binding sites for a single RNA sequence. The backend predictor of the above webservers are non-deep learning-based methods, which are proved to be inferior to deep learning-based methods for predicting RBP binding sites [18]. Moreover, no online webserver is currently available for predicting RBP binding sites on circRNAs.

However, to date, there is no online webserver available for predicting RBP binding sites on both linear and circular RNAs using deep learning. Most published approaches for predicting RBP binding sites only provide source code with different input data format, like GraphProt, our developed iDeepS and CRIP, their dependency is difficult to configure due to frequent update of deep learning framework, like TensorFlow. In addition, for deep learning-based approaches, the training of models is very time-intensive and computationally intensive. Thus, it is imperative to develop an easy-to-use webserver to integrate the state-of-the-art prediction methods for predicting RBP binding sites on RNAs and cover as many RBPs as possible. RBPsuite holds a broad application potential, it can be used to expand our knowledge about RBP binding RNAs, e.g. identifying interactions between RNA regions of SARS-COV-2 and human proteins. In addition, RBPsuite may be used to investigate the effect of mutations on RNA-protein binding sites, we can use RBPsuite to predict binding scores for an RNA sequence and a mutated RNA sequence, then check whether the mutation will greatly decrease the binding score to determine the effect of this mutation.

We implement an online webserver RBPsuite for predicting RBP binding sites on full-length linear and circular RNAs from sequences alone. For the linear RNAs, the server predicts the RBP binding scores using our updated iDeepS, which is retrained on binding RNA targets of 154 RBPs derived from ENCODE. For circRNAs, RBPsuite predicts the RBP binding scores using our developed CRIP. RBPsuite first breaks a full-length input sequence into multiple segments of 101 nucleotides without overlap, then outputs the scores between the segments and the chosen RBP. RBPsuite further detects the verified motifs on the predicted binding segments and visualizes the score distribution within the input sequence.

### Implementation

#### Collected datasets

We downloaded peaks of 154 RBPs of K526 and HepG2 through eCLIP-seq from ENCODE corresponding to human genome hg19 version. These narrow peaks were produced by the eCLIP-seq Processing Pipeline v2.0 of ENCODE [19]. To prepare the positive and negative RBP binding training data sets, several steps were processed. 1) We merge the peaks files of one RBP. It should be noted that some studies [20] used the intersection of the bed files to obtain a set of most probably peaks. 2) We select regions overlapped with reference gene by intersectBed of bedtools [21]. 3) The gene overlapped regions are extended to 101 nts in upstream and downstream centering at the read peaks, and we got the positive regions of RBPs. 4) Negative RBP binding regions were produced by implementing shuffleBed of bedtools, these negative sites are those regions without any peak located from the same gene of each peak. 5) The fasta files of positive and negative regions were retrieved by fastaFromBed of bedtools. To save the training time, for each RBP, we only keep 60,000 positive sites and 60,000 negative sites if the extracted positive and negative samples are more than 60,000, respectively. Otherwise we use all the extracted samples for this RBP.

For circRNAs, we use the trained models of 37 RBPs on the benchmark dataset of CRIP [14]. For each RBP, the number of training circRNAs (bound and non-bound) is different, they range from 992 to 40,000. Each circRNA is also a sequence segment of a size 101. More details are given in Table 1. All the collected benchmark datasets for linear and circular RNAs are freely available at http://www.csbio.sjtu.edu.cn/bioinf/RBPsuite/.

**Table 1:** The details of training and independent test sets. Each RBP has one training set and one test set, the number is the average across all RBPs

RNA type	# of RBPs	Positive data of each RBP	Negative data of each RBP
Linear RNAs	154	Training: 44,119 Independent test: 11,030	Training: 44,119 Independent test: 11,030
Circular RNAs	37	Training: 3680 Independent test: 920	Training: 3680 Independent test: 920

In addition, we downloaded verified motifs of RBPs from CISBP-RNA [22]. In total, we obtain verified motifs for 43 RBPs, which are further scanned against the sequence segments using FIMO in MEME suite [23] with *p*-value < 0.01.

#### Algorithms in RBPsuite

**Fig. 1:**
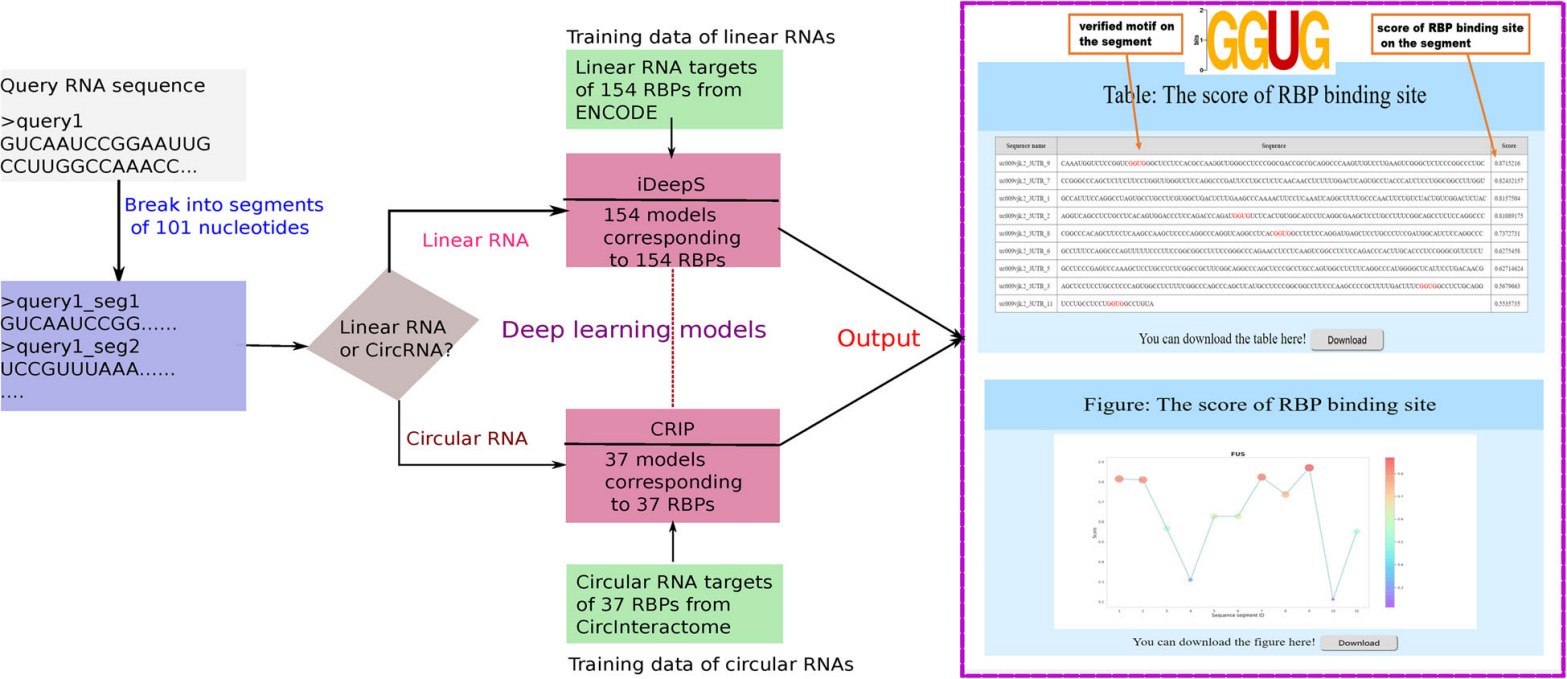
The workflow of RBPsuite webserver. RBPsuite first breaks the full-length sequence into segments of 101 nucleotides. For linear RNAs, the binding scores of individual segments are calculated by iDeepS. For circRNAs, the binding scores of individual segments are calculated by CRIP. The output page gives the binding scores for each segment and identified motifs on the segment, and also the score distribution of RBP binding sites within the input sequence

In RBPsuite, there are two deep learning-based methods: the updated iDeepS for linear RNAs, and CRIP for circRNAs. Both methods use hybrid deep models. The full picture of RBPsuite is illustrated in Fig. 1.

#### Updated iDeepS for predicting RBP binding sites on linear RNAs

Here we did some modification on the encoding schema of sequence and structure in original iDeepS. The original iDeepS encodes the sequence and structure into two individual one-hot encoded matrices and it searches sequence and structure motifs in parallel using CNNs and LSTMs, instead of combining structure and sequence features for the same motif. The structure motifs are independent from the sequence motifs, structural context may not be added. Thus, we add structure context into the motif identification to develop an updated iDeepS using an extended alphabet as used in pysster [11]. It first encodes the sequence and structure into a one-hot encoded matrix with an extended alphabet. A given RNA sequence consists of an alphabet (A, C, G, U) and the structure consists of an alphabet (F, T, I, H, M, S), we obtain an extended alphabet of a size 4*6 = 24, this extend alphabet consists of [24] with an index from 0 to 23. Then the newly one-hot encoded matrix is fed into a CNN and a LSTM to extract high-level features, which are inputted into two fully connected layers to predict RBP binding sites on linear RNAs. Here RNAshapes [24] is used to predict the abstract secondary structures from RNA sequences.

#### CRIP for predicting RBP binding sites on circRNAs

Considering that the interacting patterns of RBP-binding circRNAs are different from those of linear RNAs, the trained models on linear RNAs cannot generalize well to circRNAs. In addition, circRNAs are more structurally constrained than linear RNAs that have free ends and various secondary structure. Thus, we propose a deep learning based method CRIP for specially predicting RBP-binding sites on circRNAs [14] from sequences alone. CRIP first encodes the sequence into one-hot encoded matrix using a stacked codon-based encoding scheme, then the encoded matrix is fed into a hybrid deep learning architecture with a CNN and a biLSTM to predict RBP binding sites on circRNAs.

#### Detecting binding motifs using MEME

To further provide the support evidence for predicted binding sites, we use FIMO [25] in MEME [23] to scan the occurrence of verified motifs on the predicted binding segments. To this end, we first collect the verified motifs of RBPs from CISBP-RNA database [26]. Then for a given RBP, we use FIMO to scan its known motif against those segments with a predicted score > 0.5 by RBPuite, the *p*-value threshold 0.01 is used and other parameters are defaulted values.

#### Development environment

iDeepS and CRIP in RBPsuite are implemented under the TensorFlow framework in Python. Given a full-length RNA sequence, it will break the sequence into multiple segments of 101 nts (used by iONMF [27] and our previous iDeep) without overlap, if the input sequence or the remaining sequence is shorter than 101 nt, we pad it to a length of 101 using ‘N’ as another 101 nt-long segment. Then these generated segments are fed into the iDeepS and CRIP to give the binding scores between individual segments and a specified RBP.

The frontend of RBPsuite webserver uses JQuery framework of JavaScript and Ajax technology to implement asynchronous loading. The backend uses PHP to call shell and python scripts. For the visualization, RBPsuite directly uses Matplotlib to display the results.

## Results and discussion

### Performance of RBPsuite webserver

**Fig. 2:**
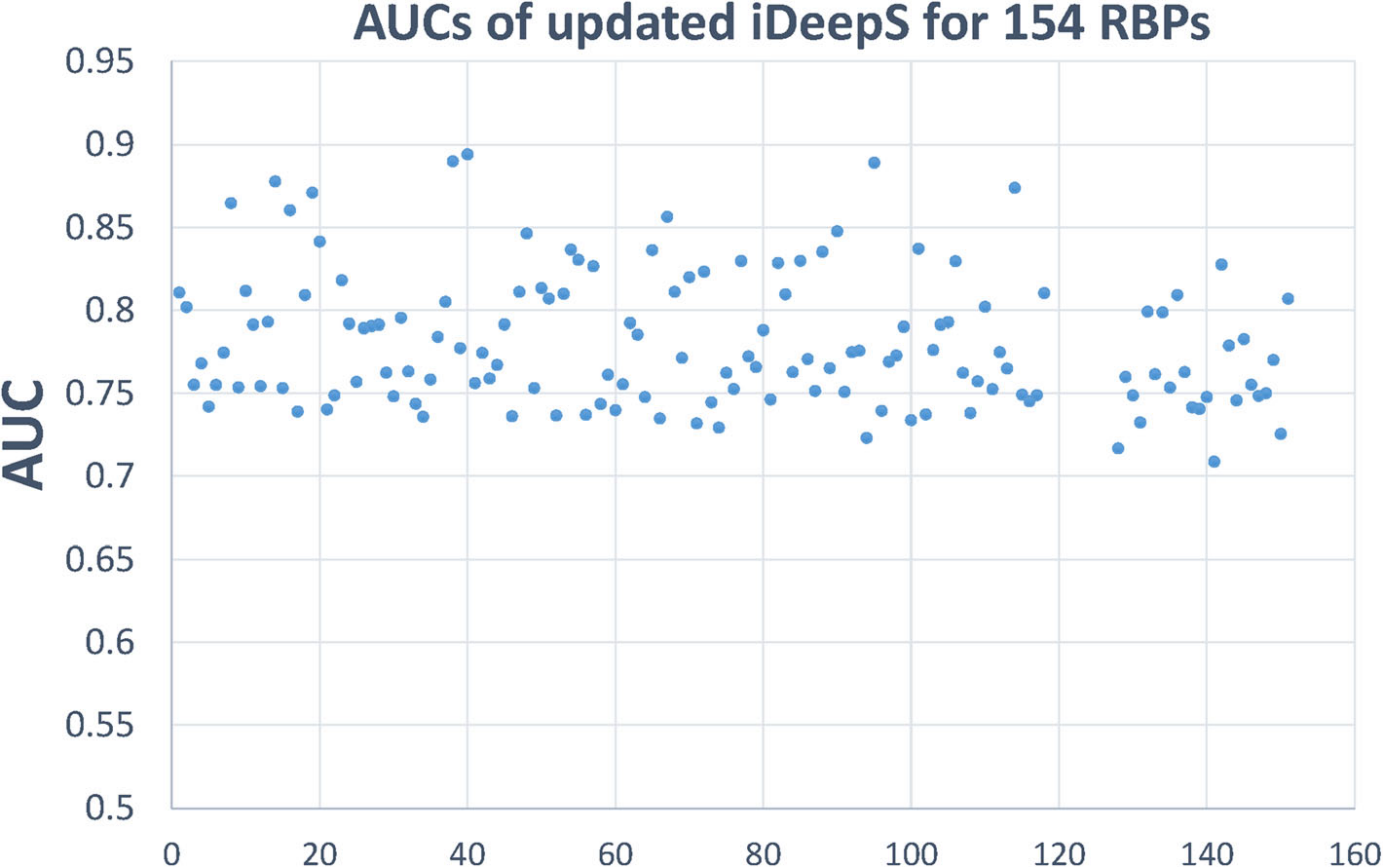
The AUCs of the updated iDeepS for linear RNAs on 154 RBPs

We first evaluate the updated iDeepS on the original benchmarked dataset with 31 experiments [8], iDeepS yields an average AUC of 0.85 across 31 experiments, which is close to the original iDeepS. Our previous study [10] demonstrates that iDeepS is superior to DeepBind and GraphProt. In addition, the independent study [12] demonstrates that iDeepS performs similarly to the latest DeepCLIP with a similar network architecture on the benchmark dataset from GraphProt. For linear RNAs, iDeepS in RBPsuite yields an average AUC of 0.781, precision of 0.673, sensitivity of 0.802 and specificity of 0.591 across 154 RBPs on the independent test set. As shown in Fig. 2, the AUCs for 154 RBPs are all greater than 0.7. We also retrain CRIP on the circRNA benchmark set, CRIP yields an average AUC of 0.878, a precision of 0.798 and a sensitivity of 0.813, across 37 RBPs.

### Input of RBPSuite

The RBPsuite accepts a full-length RNA sequence or a sequence file in FASTA format as the input. It also accepts batch input with multiple RNA sequences. The length of each RNA sequence is not limited, but all sequences have to be nucleotides ‘ACGUT’.

In addition to the input sequence, users need specify the RNA type ‘Linear RNA’ or ‘Circular RNA’, which determines which computational method will be used for predicting the RBP binding sites. If the RNA type of input RNA is unknown, WebCircRNA is recommended for assessing the circRNA potential. According to the potential score estimated by WebCircRNA [28], users can choose the RNA type. After choosing the RNA type, the users are required to choose a model. ‘Specific model’ predicts the binding scores between the input RNA and the chosen RBP using the models trained on the RNA targets of the chosen RBP. ‘General model’ predicts the binding scores between the input RNA and all RBPs with trained models, and the number of RBPs is 154 and 37 for linear RNAs and circRNAs, respectively.

### Output of RBPSuite

When the job is finished, the prediction results will appear on the results page. For each job, a job-ID will automatically be assigned, users can use the job-ID to track the job progress and retrieve the results later. For the chosen RBP, the result page consists of one sortable table listing the segments with binding score greater than 0.5 and a score distribution figure of all segments according to their positions within the input RNA. If there are verified motifs for the RBP, the motifs on the segments in the result table are marked in red. All the prediction results are downloadable in the result page. The expected runtime of predicting binding sites of a specific RBP on a linear RNAs and a circRNAs using RBPsuite for sequences with different lengths are listed in Table 2. For longer sequences, iDeepS for linear RNAs takes longer time than CRIP for circRNAs since it first needs run the structure prediction.

**Table 2:** The expected runtime of predicting binding sites of a specific RBP on a linear RNA and a circRNA using RBPsuite for sequences with different lengths

RNA type	Sequence length	Time(s)
Linear RNA	1000	5.62
	10,000	15.43
	100,000	115.59
circRNA	1000	9.07
	10,000	9.79
	100,000	20.26

**Fig. 3:**
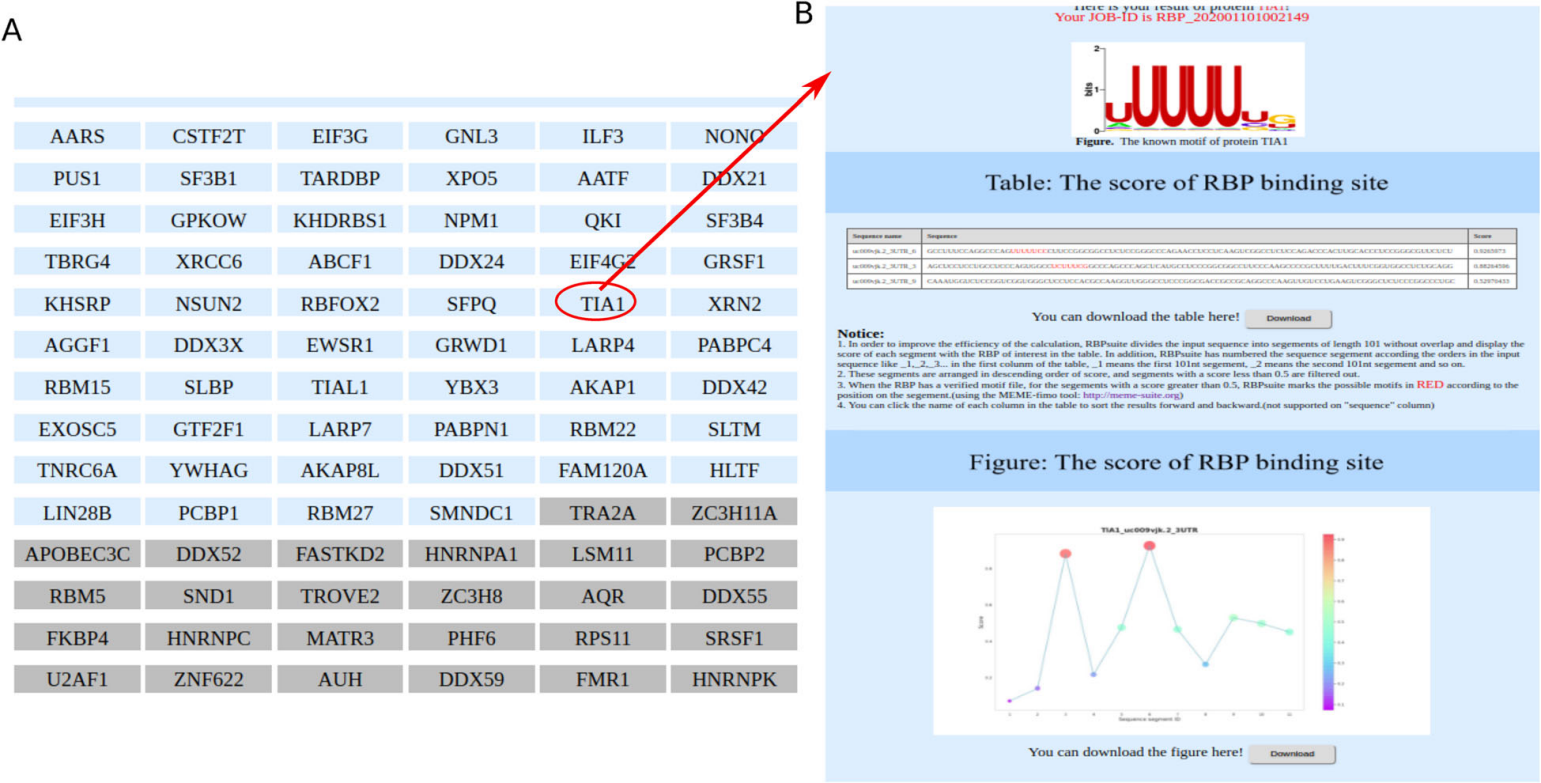
The output of RBPsuite for general model, clicking a protein of interest to see the detailed results for this protein. In the table, the detected motif on the predicted binding site is marked in red

For general model, RBPsuite will predict binding scores of all available RBPs for the segments of the input sequence, as shown in Fig. 3a. Users can click the RBP of interest to see the predicted RBP binding sites of this RBP on the input sequence (Fig. 3b).

### Case study

**Fig. 4:**
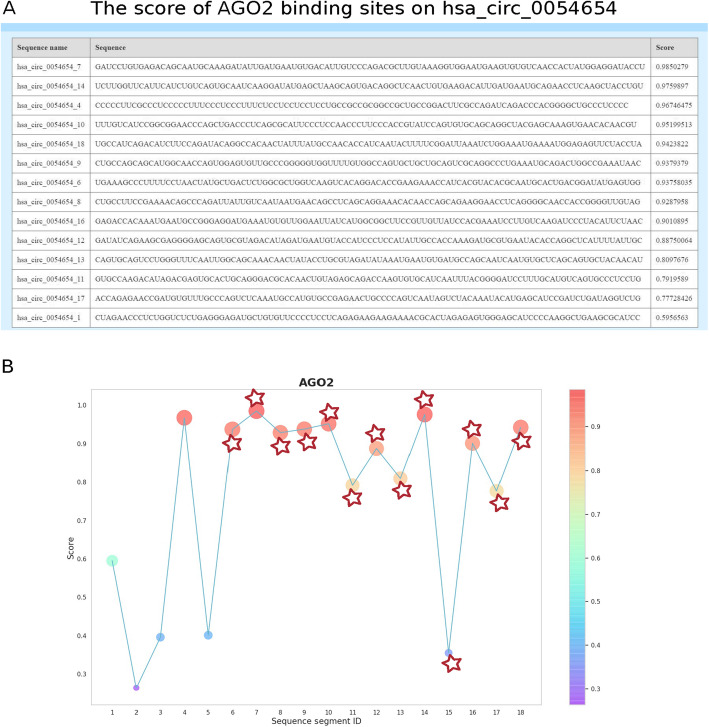
The results of RBPsuite for predicting AGO2 binding sites on hsa_circ_0054654. A) The 101 nt segments of hsa_circ_0054654 with a binding score greater than 0.5. B) The score distribution of 18 segments from hsa_circ_0054654, where the star corresponds to the verified binding sites derived from CLIP-seq read peaks

Here we use RBPsuite to predict RBP binding sites on full-length RNAs. We use circRNA hsa_circ_0054654 as an example. hsa_circ_0054654 has a length of 1821 nts, and it has 13 AGO2 binding sites with the CLIP-seq peaks without overlap. RBPsuite first breaks the hsa_circ_0054654 sequence into 18 segments, which are predicted to be 14 AGO2 binding sites with a score cutoff 0.5, as shown in Fig. 4a. Of the 14 predicted binding sites, 12 are the segments with verified binding sites locating on, only one segment with verified binding site is not detected by RBPsuite (Fig. 4b), where star is the verified binding sites of AGO2. As shown in Fig. 4b, only two segments are wrongly predicted as AGO2 binding sites, one has a low predicted score below 0.6.

### Future development

In RBPSuite, we use FIMO in the MEME tool to detect verified motifs from CISBP-RNA database within the segments of the input RNA sequences. In iDeepS, we can extract binding motifs from the learned parameters of the kernels of CNNs. However, these detected motifs are still not experimentally verified. Another future direction of RBPSuite is to apply integrate gradient [29] to highlight key nucleotides for binding to RBPs, instead of limiting to the verified binding motifs. For example, TF-modisco [30] uses the attribution maps generated by integrated gradients to extract summary motifs.

One limitation of RBPsuite is that it can only predict binding targets for those RBPs with a certain number of verified binding targets. It is estimated there exist over 1000 human RBPs [31], whose binding targets may be screened in future. Thus, we will update RBPsuite to cover more RBPs with more advanced computational methods. Another solution is that transferring models from RBPs with similar binding preference to the RBP with limited verified targets, as done in beRBP [32], which is able to predict binding sites for any RBPs. In addition, RBPsuite predicts a 101 nt-long segment locating the RBP binding site but still cannot locate the exact binding nucleotides within this segment. More advanced computational methods will also be added to the existing framework in future. We expect to update RBPsuite to be able to locate the exact binding nucleotides on RNAs.

## Conclusions

In this study, we implement an online webserver RBPsuite for predicting RBP binding sites on linear and circular RNAs based on deep learning. RBPsuite integrates two deep learning algorithms iDeepS and CRIP, which predict RBP binding sites on linear RNAs and circRNAs, respectively. RBPsuite is able to predict binding linear RNAs for the largest number of RBPs, and is the first deep learning-based webserver for this task. The RBPsuite accepts RNA sequence as the input and gives the scores of 101 nt segments broken from the input RNA sequence. In addition, RBPsuite further detects the verified motifs on the segments to give more evidence for supporting the binding segments. The prediction performance on the independent test set and a case study both demonstrate the effectiveness of RBPsuite.

## Availability and requirements

**Project name:** RBPsuite

**Project home page:** http://www.csbio.sjtu.edu.cn/bioinf/RBPsuite/

**Operating system(s):** Platform independent

**Other requirements:** Google chrome, Safari and Firefox

**License:** Apache License 2.0

**Any restrictions to use by non-academics:** Licence needed

## Data Availability

The data and online webserver is available at http://www.csbio.sjtu.edu.cn/bioinf/RBPsuite/.

## Abbreviations

AUC: Area under the ROC curve
RBPs: RNA binding proteins
circRNA: Circular RNA
HMM: Hidden Markov Model
LSTM: Long short term memory network
CNN: Convolutional neural network
PWM: Position weight matrix
ROC: Receiver operating characteristic
